# Challenges of Estimating the Annual Caseload of Severe Acute Malnutrition: The Case of Niger

**DOI:** 10.1371/journal.pone.0162534

**Published:** 2016-09-08

**Authors:** Hedwig Deconinck, Anaïs Pesonen, Mahaman Hallarou, Jean-Christophe Gérard, André Briend, Philippe Donnen, Jean Macq

**Affiliations:** 1 Institut de recherche santé et société, Université catholique de Louvain, Brussels, Belgium; 2 École de médecine, Université catholique de Louvain, Brussels, Belgium; 3 École de santé publique, Université libre de Bruxelles, Brussels, Belgium; 4 Save the Children, Niamey, Niger; 5 Tampere Centre for Child Health Research, University of Tampere and Tampere University Hospital, Tampere, Finland; 6 Department of Nutrition, Exercise and Sports, Faculty of Science, University of Copenhagen, Frederiksberg, Denmark; Kenya Medical Research Institute - Wellcome Trust Research Programme, KENYA

## Abstract

**Introduction:**

Reliable prospective estimates of annual severe acute malnutrition (SAM) caseloads for treatment are needed for policy decisions and planning of quality services in the context of competing public health priorities and limited resources. This paper compares the reliability of SAM caseloads of children 6–59 months of age in Niger estimated from prevalence at the start of the year and counted from incidence at the end of the year.

**Methods:**

Secondary data from two health districts for 2012 and the country overall for 2013 were used to calculate annual caseload of SAM. Prevalence and coverage were extracted from survey reports, and incidence from weekly surveillance systems.

**Results:**

The prospective caseload estimate derived from prevalence and duration of illness underestimated the true burden. Similar incidence was derived from two weekly surveillance systems, but differed from that obtained from the monthly system. Incidence conversion factors were two to five times higher than recommended.

**Discussion:**

Obtaining reliable prospective caseloads was challenging because prevalence is unsuitable for estimating incidence of SAM. Different SAM indicators identified different SAM populations, and duration of illness, expected contact coverage and population figures were inaccurate. The quality of primary data measurement, recording and reporting affected incidence numbers from surveillance. Coverage estimated in population surveys was rarely available, and coverage obtained by comparing admissions with prospective caseload estimates was unrealistic or impractical.

**Conclusions:**

Caseload estimates derived from prevalence are unreliable and should be used with caution. Policy and service decisions that depend on these numbers may weaken performance of service delivery. Niger may improve SAM surveillance by simplifying and improving primary data collection and methods using innovative information technologies for single data entry at the first contact with the health system. Lessons may be relevant for countries with a high burden of SAM, including for targeted emergency responses.

## Introduction

Prevalence estimates suggest that severe acute malnutrition (SAM) affects 16 million children worldwide [1] and kills over half a million children annually [2]. These prevalence figures, derived from cross-sectional surveys, may not reveal the true picture of the SAM burden.

Children with SAM have a high risk of death and require intensive medical care if they are detected late or have developed medical complications [3]. Until 2000, children with SAM were managed as inpatients, with low coverage and high case-fatality. In the past decade, improved outpatient treatment protocols and the innovation of ready-to-use therapeutic foods facilitated scale-up of decentralised management of SAM in primary health care. This approach is now being implemented in about 80 countries [4]. While integration of the management of SAM into child healthcare is being promoted, its scale-up in high-burden, low-income countries such as in Niger faces many challenges, including weak health service systems [5, 6].

Reliable estimates of SAM burden and caseload for treatment are needed for policy decisions and for planning, implementing and evaluating services in the context of competing public health priorities and limited resources. These estimates are considered a major step toward the development of cost-effective health interventions [7]. A 2013 review of SAM interventions in Niger [8] highlighted weaknesses in the methods used to estimate caseload for treatment at the start of the year and to report case detection and admissions during the year. These weaknesses are expected to affect the quality and effectiveness of care. This paper compares the reliability of estimating the annual SAM caseload for treatment of children 6–59 months of age in Niger at the start of the year with the reliability and accuracy of estimating the annual incidence of case detection and admission at the end of the year. It also discusses factors that account for differences in estimated SAM caseload, incidence and coverage.

## Methods

### Study design

This study used secondary data collected during an evaluation of SAM interventions in 2013. Data were extracted from survey reports and various databases from two health districts, Aguie in Maradi Region and Matameye in Zinder Region, for 2012 and the country overall for 2013. National nutrition surveys were conducted by the National Institute of Statistics of the Government of Niger. District nutrition and coverage surveys were conducted by the respective district health offices with support from Save the Children. Data from the various acute malnutrition surveillance systems were collected and managed by the respective district health offices, the Directorate of Nutrition, and the Directorate of Statistics of the Ministry of Public Health with support from the United Nations Children’s Fund. Annual burden and caseload estimations of SAM were calculated using data and formulas applied in Niger. Coverage was extracted from survey reports, and incidence from three SAM surveillance systems. Data from the two districts and the country overall were used to compare the trend of estimates across information systems, settings, and time.

### Study definitions and data sources

#### SAM definition and indicators

SAM is defined by either weight-for-height below (<) minus 3 z-score standard deviations of the median value (WHZ <-3) of the 2006 WHO Child Growth Standards or mid-upper arm circumference (MUAC) less than (<) 115mm or the presence of nutritional oedema [9, 10]. (Moderate acute malnutrition [MAM] is defined by either WHZ equal to or above [≥] -3 and <-2 or MUAC between ≥115mm and <125mm [9, 10].)

#### Diagnosing and recording SAM

Children detected with SAM in the community by MUAC or oedema were referred for treatment to the closest primary care facility where treatment was offered. Health posts with no nurses on staff did not offer SAM treatment, but community health workers or lay health workers (volunteers) measured children with suspected SAM. Results were marked on a SAM-specific tally sheet, and children with either WHZ <-3 or MUAC <115mm or oedema were referred for treatment. At the health centres (and health posts with nurses on staff), volunteers measured children with suspected SAM. Children with either WHZ <-3 or MUAC <115mm or oedema received a clinical examination for the presence of oedema, appetite and co-morbidities. Results for children with either WHZ <-3 or MUAC <115mm or oedema were written on scrap paper, then marked on a SAM-specific tally sheet, then copied in the SAM-specific outpatient register, children’s outpatient treatment cards, health cards if available, therapeutic food ration cards kept by carers and in case of referral to hospital for inpatient treatment of complications, marked on referral slips. At the hospital, volunteer, auxiliary and clinical health workers collaborated to measure the children on admission, perform a medical examination, monitor progress daily until complications resolved and refer the children back to primary care to continue treatment as outpatients. Results were recorded in the SAM-specific inpatient registers, SAM-specific inpatient treatment cards, regular hospital registers and medical and drug record cards. Children admitted for treatment were given SAM-specific numbers that were copied into the different records at the various levels of service delivery.

#### Counting new SAM cases

Three different surveillance systems provided counts of new SAM cases, or incidence. First, the notifiable disease surveillance system *(Maladies à déclaration obligatoire*, *or MDO)* of the national health information system provided weekly numbers of new SAM (and MAM) cases and deaths in every health facility in the country. The responsible health workers used data from either the SAM-specific tally sheets or registers to fill out the *MDO* reporting sheet, which was shared weekly with the district health offices. Data were entered into the health information system database at the district level and shared and amalgamated at the regional and national level. Second, a *Monthly Report* system introduced in 2005 as part of SAM-specific monitoring and evaluation provided detailed monthly information on case admissions and exits and stock use from every site providing SAM (and MAM) treatment. Health workers used the SAM-specific registers to fill out the monthly reporting sheets. Reports were shared with the district health offices monthly. Data were entered into the SAM-specific database at the health district level and shared and amalgamated at the regional and national level. Third, another weekly surveillance system, *Scaling Up*, was introduced in 2007 to provide instant and reliable reporting of case admissions and exits and stock use from every site providing SAM (and MAM) treatment. Health workers used the SAM-specific registers to fill out the weekly reporting sheets. Reports were shared with the health districts, regions and central office weekly. Data were entered into a different SAM-specific database at the health district level and shared and amalgamated at the regional and national level. This case study extracted data from the databases [11–13] of SAM detection and admissions for the two health districts in 2012 and for Niger overall in 2013.

#### Caseload of SAM

For the purpose of this paper, burden of SAM refers to the overall number of children with SAM in the population, and SAM caseload refers to the number of children with SAM who effectively access and take up treatment (or number of admissions). The annual burden (Eq 1) and caseload (Eq 2) were calculated as follows:

Annual burden of SAM = Prevalent SAM cases in the population at the start of the year + New SAM cases during the year (1)

Annual caseload of SAM = Prevalent SAM cases in treatment at the start of the year + New SAM cases admitted for treatment during the year (2)

#### Estimating SAM caseload prospectively

Because SAM incidence is not known, an indirect method was used to calculate the annual expected SAM burden and caseload. In stable populations, for uncommon illnesses, prevalence is a function of incidence and duration of untreated illness (Eq 3) [14]. Thus, if prevalence and duration of illness are known, incidence (Eq 4), and the incidence conversion factor (Eq 5) can be estimated. The period *t* for which the incidence was calculated was 12 months (Eq 6). Prevalence of SAM defined by either WHZ <-3 or presence of oedema was derived from surveys.

Prevalence = Incidence * Average duration of untreated illness  (3)

Incidence = Prevalence * t / Average duration of untreated illness (4)

Incidence conversion factor = t / Average duration of untreated illness  (5)

Annual incidence = Prevalence * 12 months (or 365 days) / Average duration of untreated illness  (6)

This case study retrieved SAM prevalence data from national nutrition survey reports [15, 16] and nutrition survey reports from Aguie and Matameye health districts [17, 18] for the respective years applying the Standardized Monitoring and Assessment of Relief and Transition (SMART) method [19]. Duration of untreated SAM illness is impossible to study for ethical reasons but has been estimated from historical data or modelled from recent data. Garenne et al. (2009) estimated the duration of an untreated episode of SAM from historical datasets at 7.5 months [20]. The 7.5 months of duration of SAM illness and 12-month period yielded an incidence conversion factor of 1.6 (12 months divided by 7.5 months equals 1.6). This conversion factor has been promoted globally to estimate annual SAM incidence from prevalence [21]. Isanaka et al. (2011) used empirical data to model the duration of untreated SAM illness in Maradi District in Maradi Region, Niger, and found it to be 45 days, resulting in an incidence conversion factor of 8.1 (365 days divided by 45 days equals 8.1) [22]. Health actors in Niger had noted that the proposed 1.6 conversion factor systematically underestimated the annual burden and adjusted it for their own use to 2.0, 2.3 or 2.5 [8]. This case study used the different incidence conversion factors to compare prospective annual caseload estimates with incident cases. The expected annual burden (Eq 7) differed from the expected annual caseload (Eq 8) because not all children with SAM were expected to access and take up treatment. The caseload calculation therefore adjusted for 50% coverage, acceptable coverage in rural areas compared with international standards [23]. In both equations, prevalence was defined by either WHZ <-3 or the presence of oedema at the time of the survey, using SAM prevalence data from the previous year for the coming year.

$$\begin{array}{l} \text{Expected annual burden of SAM }\\ =\text{ Child population }6\text{-}59\text{ months }*\;(\text{Prevalence }+\text{ Incidence})\;=\text{ Child population }6\text{-}59\text{ months }*\;(\text{Prevalence }*\;[1\;+\text{ Incidence conversion factor}]) \end{array}$$ (7)

$$\begin{array}{l} \text{Expected annual caseload }(\text{annual burden adjusted for }50\%\text{ coverage})\;\\ =\text{ Child population }6\text{-}59\text{ months }*\text{ 0.5 }*\;(\text{Prevalence }*\;[1\;+\text{ Incidence conversion factor}]) \end{array}$$ (8)

#### Coverage

Contact coverage, defined by the proportion of children with SAM in the population receiving treatment, is a key indicator of service performance and compares with international standards [23]. Different methods were used in Niger to estimate contact coverage of SAM.

First, cross-sectional coverage survey methods used local area sampling to assess contact coverage in health districts and large area sampling to assess contact coverage country-wide [24]. The surveys provided estimates of point coverage (proportion of children with SAM in the population receiving treatment) and period coverage (proportion of children with SAM and recovering from SAM in the population receiving treatment) that evaluated different aspects of service performance because of the additional time factor of recovery. Contact coverage of SAM was also estimated by cross-sectional nutrition surveys, with low precision. This case study retrieved the national coverage rate [25] and the Matameye Health District coverage rates from coverage surveys [26]. The coverage survey conducted in Aguie Health District in 2012 studied barriers to service access but did not yield a coverage rate [27]. In the coverage surveys, SAM was identified by either MUAC <115mm or presence of oedema. Second, an indirect method was used to measure whether SAM intervention targets were reached. Admissions at the end of the year were compared with the expected target caseload at the start of the year (Eq 9). The target caseload of SAM was derived from SAM prevalence and adjusted for 50% coverage. The numerator was derived from admissions of SAM defined by either WHZ <-3 or MUAC <115mm or presence of oedema. The denominator was derived from nutrition surveys, in which SAM was defined by either WHZ <-3 or presence of oedema.

$$\begin{array}{l} \text{Proportion of covered need of SAM treatment in children }6\text{-}59\text{ months at the end of the year }\\ =\text{ Number of children }6\text{-}59\text{ months with SAM admitted for treatment during the year }/\text{ Expected annual caseload at the start of the year } \end{array}$$ (9)

#### Population figures

This case study used the same population figures of children 6–59 months of age as the annual health information system used to report Niger health indicators [28, 29].

## Results

Annual caseloads are presented from prospective estimations and retrospective counts of incident cases from three surveillance systems, with their respective incidence rates and conversion factors. Because coverage estimates are used in prospective estimations of caseload, available contact coverage results of the respective sites are presented first.

### Contact coverage

Contact coverage of SAM in Matameye Health District was good (>50%) to low (<30%) according to international standards [23] (Table 1) [26]. Good coverage still meant that half of all children with SAM did not access treatment. Trends in coverage fluctuated and decreased significantly in 2013 compared with 2012. Contact coverage of SAM in Niger overall was low, indicating that three out of four children with SAM did not access treatment [25].

**Table 1:** Contact coverage of severe acute malnutrition in children 6–59 months of age estimated by coverage survey methods.

Site	Contact coverage of severe acute malnutrition (% and 95% confidence intervals)			
	2010	2011	2012	2013
**Matameye**	34.9 (26.3–44.2)	46.2 (36.8–55.8)	53.3 (45.0–61.2)	36.9 (30.4–43.8)
**Niger**	/	19^a^	/	/

^a^Confidence interval not available.

### Annual caseload

Table 2 compares SAM cases derived from indirect and direct methods. First, caseload was estimated by the indirect method at the start of the year from prevalence by applying an incidence conversion factor of 2 adjusted for an expected coverage of 50%, which was the usual method in Niger. SAM prevalence estimates were high for Aguie (3.0%) and Matameye (3.0%) in 2012 and for Niger overall (2.6%) in 2013, and the confidence intervals yielded wide ranges in estimated caseload. Second, the three surveillance methods (*MDO*, *Scaling Up and Monthly Report*) (direct method) counted incident cases over the year. Caseload numbers at the end of the year showed variation across the three methods in the three sites (Aguie, Matameye and Niger overall). For example, the weekly *MDO* detected more cases than the weekly *Scaling Up* and *Monthly Report* reported admissions in Aguie and Niger overall, but detected only half the number of admitted cases in Matameye. The *Monthly Report* under-reported cases in Aguie but over-reported cases in Matameye in comparison to weekly reported cases. Third, the indirect method expected considerably fewer cases at the start of the year than the actual number of admissions over the year. The upper level predictions of expected cases were less than half the numbers detected or admitted. For example, for Niger overall, the maximum number of expected cases at the start of the year (156 347) was 2.6 times smaller than the number of cases admitted at the end of the year (406 327). The minimum degree of underestimation was 1.7 (11 688/6 827) for Aguie and 3.4 (21 488/6 301) for Matameye. Matameye Health District data from *Scaling Up* and national amalgamated data from the *Monthly Report* were not available.

**Table 2:** Annual caseload of severe acute malnutrition estimated at the start of the year from prevalence and counted at end of year from three surveillance systems.

Site	Child population6–59 months^a^	SAM^b^ prevalence rate	SAM^b^ cases estimated at start of year^d^	SAM^c^ cases counted at end of year		
				*MDO*^e^	*Scaling Up*	*Monthly Report*
	(number)	(% and 95% confidence interval)	(number and 95% confidence interval)	(number)	(number)	(number)
**Aguie**	91 019	3.0 (1.8–5.0)	4 097 (2 457–6 827)	17 151	16 164	11 688
**Matameye**	75 021	3.0 (1.6–5.6)	3 377 (1 800–6 301)	11 956	/	21 488
**Niger**	3 362 293	2.6 (2.2–3.1)	131 130 (110 955–156 347)	409 367	406 327	/

^a^Child population estimations for the respective years [28, 29].

^b^Severe acute malnutrition (SAM) defined by either WFZ <-3 or presence of oedema.

^c^SAM defined by either WHZ <-3 or MUAC <-115mm or presence of oedema.

^d^Caseload estimated from prevalence, incidence factor 2 and 50% coverage, the usual method used in Niger.

^e^*Maladies à déclaration obligatoire*.

### Incidence and conversion factor

Table 3 shows SAM incidence rates and incidence conversion factors obtained by the three surveillance systems. Incidence rates were obtained by dividing the number of admissions by the average mid-year child populations (Table 2).

- In Aguie, the *MDO* reported that one out of five children was detected with SAM. *Scaling Up* reported one out of six children as admitted for treatment for an episode of SAM, and the *Monthly Report* reported one out of eight.
- In Matameye, the *MDO* reported that one out of six children was detected with SAM, and the *Monthly Report* reported that one out of four children was admitted for treatment for an episode of SAM.
- In Niger overall, one out of eight children was detected and admitted for an episode of SAM.

**Table 3:** Annual incidence rates and incidence conversion factors of SAM obtained from three surveillance systems.

Site	SAM^a^ incidence rate (%)			SAM^a^ incidence conversion factor (number)		
	MDO^b^	Scaling Up	Monthly Report	MDO	Scaling Up	Monthly Report
**Aguie**	18.8	17.7	12.8	6.3	5.9	4.3
**Matameye**	15.9	/	28.6	5.3	/	9.5
**Niger**	12.2	12.1	/	4.7	4.6	/

^a^Severe acute malnutrition (SAM) defined by either WHZ <-3 or MUAC <115mm or presence of oedema.

^**b**^*Maladies à déclaration obligatoire*.

The SAM incidence conversion factors by site and surveillance system were obtained by dividing incidence by prevalence. The incidence conversion factors yielded numbers that ranged from 4.3 to 9.5, two to four times higher than factor 2, which was commonly used to estimate caseloads in Niger.

## Discussion

The case study shows that the expected caseload of SAM derived from prevalence at the start of the year was lower than the actual caseload counted over the year, and uncovers differences in incidence counts. This section discusses methodological and practical challenges in providing reliable and accurate figures on SAM caseload, incidence and coverage by the various methods used in Niger.

### Challenges in estimating annual SAM caseload at the start of the year

Annual caseload was commonly analysed prospectively in Niger, as elsewhere in the world, for planning, implementation and evaluation purposes [21]. The study showed this method to be inaccurate. Reasons could be related to the unsuitability of prevalence to understand the SAM burden, the use of different SAM indicators identifying different SAM populations and the inaccuracy of figures for duration of illness, expected contact coverage and population.

First, prevalence is not an appropriate measure for an acute condition because it counts only cases at the time of the survey but misses cases that developed and died or recovered between survey intervals. Nor does prevalence capture unexpected spikes or seasonal fluctuations. Moreover, the time and context interpretation of the prevalence estimate may be lost during its applications. These are important considerations for SAM and even more so for the acute condition of oedematous SAM. Besides, surveys may use different data cleaning criteria to exclude extreme data values, which may impact on prevalence estimates. For example, one study comparing severe wasting prevalence results between survey methods found that SMART used stricter cleaning criteria, excluding three to five times more records, resulting in differences in the estimated prevalence of severe wasting between 0.4% and 3.9% [30]. In addition, relying on SAM point prevalence estimates without taking into account uncertainty intervals may give a false reassurance of accuracy [31]. Second, different anthropometric indicators were used to define SAM. For example, nutrition surveys reported SAM prevalence based on either WHZ or presence of oedema (WHZ + oedema cases), and when MUAC was measured, reported on SAM prevalence based on either MUAC or oedema (MUAC + oedema cases) separately. Coverage surveys detected SAM based on either MUAC or oedema (MUAC + oedema cases) but never on WHZ, while surveillance systems detected SAM based on either WHZ or MUAC or presence of oedema (WHZ + MUAC + oedema cases). Because WHZ and MUAC do not measure the same SAM phenomena [10] and SAM children are diagnosed by one indicator, different sources reporting on SAM using different indicators did not compare the same SAM populations [32]. Presence of oedema was the prime SAM diagnostic, and if negative, a MUAC <115mm or WHZ <-3 indicated SAM in individual children. Third, duration of untreated illness is not stable in time, between individuals or across populations and therefore should be used with caution to estimate incidence from prevalence. The universally promoted conversion factor of 1.6 was estimated from historical longitudinal data sets [33] that measured incidence of SAM defined by low MUAC measured at 3- to 6-month intervals. Duration of illness obtained from these data sets has shortcomings. Broad MUAC measurement intervals may not have captured all new cases that developed and died or recovered and therefore underestimated incidence. Also, the epidemiological and child healthcare context has changed since these studies. Further, the study did not include oedematous SAM. Another conversion factor of 8.1 was obtained by modelling duration of untreated illness based on data from Niger [22]. This modelling also applied a range of assumptions that may differ from practice. The range of the two conversion factors is wide, resulting in great differences in annual estimations. Results from this case study found that the conversion factor obtained through modelling was more realistic. Fourth, prospective estimates of SAM caseload were adjusted for 50% coverage, the benchmark for good contact coverage in rural settings [23]. Such high coverage has rarely been obtained in Niger or elsewhere in the world except in high-resource contexts [34]. As such, the annual expected caseload at the start of the year was overestimated, and its use to evaluate whether SAM interventions met the targets set at the start of the year was inappropriate. Finally, because of high annual population growth (4.02% in 2013) [35], the delayed release of the 2012 census data and population movements, health actors in the country used different population figures, further affecting the accuracy of caseload estimates.

### Challenges in counting SAM incident cases

Differences and discrepancies in incidence obtained by the three surveillance methods raised questions about the quality of the primary data; performance of health actors in measuring, recording and reporting SAM indicators; and the different purposes of the surveillance systems.

First, detection of SAM depended on the quality of the anthropometric equipment and skills to measure MUAC, weight and height accurately; verify the WHZ indicator in the child standards tables for girls and boys combined or separated (two different tables were in use); and check for the presence of oedema. The same measurement results were recorded three to four times in different information systems. Random verification of records often revealed physiologically impossible anthropometry. Second, a team of volunteers, trained, supervised and motivated by non-governmental organisations, usually measured children with suspected SAM at health facilities. Volunteers were generally dedicated lay health workers. Spot checks revealed that a combination of limited skills, the complexity of measuring and recording results in a language different from their mother tongue and the usually hectic and overcrowded work environment influenced their performance. Shifting diagnostic tasks to volunteers therefore had mixed results. Volunteers reduced the workload of health workers, but they hampered improvements of identified weaknesses in the surveillance system because they were not formal staff. Third, health workers faced difficulties applying the different indicators to report detected SAM cases in the weekly *MDO* and to report admitted cases in the weekly *Scaling Up* and *Monthly Report*. Spot checks revealed knowledge gaps of health workers about the different meanings and purposes of the indicators and their respective surveillance systems. Moreover, health workers spent many hours filling out the weekly or monthly reporting sheets for SAM and other health programmes. Spot checks revealed some SAM reporting sheets with identical numbers, calling their reliability into question and underlining a motivational issue affecting performance. Fourth, the different databases were maintained at the district, regional and national levels of the Ministry of Public Health, with different cycles of submission and data analysis. In parallel, technical partner agencies involved in SAM interventions copied datasheets and maintained databases for monitoring and evaluation of their respective programmes, catchment areas and funding cycles. *Scaling Up* had a national reporting rate close to 80%, better than the *MDO* and *Monthly Report*, but it also received the most resources. It was indeed expected that the *MDO* would yield higher numbers of detected SAM than the surveillance systems for admissions, but this was not the case for Matameye Health District. It was also expected that the *Monthly Report* of the two districts receiving partner support would provide accurate admission numbers, which the study results could not confirm. Spot checks revealed that the organisation of primary data collection, the management of the databases and the performance of health workers differed within and across districts. The variation in implementation may have explained the erratic differences in surveillance results. Despite this, limited quality control measures were in place. Rumours hinted at children being counted several times either by attending more than one health centre or by sharing cards of ghost or replacement children, but this failed to explain the major differences in annual incidence of SAM reported. Audits of surveillance systems compared results but did not uncover why and how and in what circumstances differences in incidence results were produced.

### Challenges in estimating SAM coverage

Direct methods of SAM coverage estimation were used in Niger to evaluate the performance of SAM interventions by assessing SAM service access, uptake and retention. An indirect method was also used to evaluate retrospectively whether the target caseload had been reached. Both methods entailed challenges.

First, coverage surveys were rare events, as they were labour intensive and required expensive specific international expertise. They usually provided estimates of point coverage (proportion of children with SAM receiving treatment) and period coverage (proportion of children with SAM and recovering from SAM receiving treatment) with uncertainty intervals. The distinction between point and period coverage may be useful in the intervention context but lost its meaning when applied for other purposes. Spot checks found that the better coverage estimate of the two was usually retained. Consequently, the interpretation of direct coverage estimates may have been inaccurate. Coverage surveys conducted in Matameye Health District showed variation that may be linked to changes in resources or intervention strategy. We recommend exploring how these changes affected coverage results by a dynamic systems method, for example, the behaviour-over-time graph and realist evaluation [36, 37]. Coverage estimates from nutrition surveys were not studied, as their sampling method was not adequate to estimate point coverage with adequate precision, and they were therefore impractical or of limited value. Second, results from indirect coverage methods, comparing annual admissions at the end of the year with expected admissions at the start of the year, were not discussed. The unrealistic underrating of expected caseload estimations made the coverage estimate inappropriate. For example, this method underestimated coverage of SAM in Aguie in 2012 by four times (16 164 / 4 097). Nevertheless, the indirect coverage estimation method was still being used and promoted [21], while it should be abandoned. More sustainable alternatives to obtain SAM coverage information may be explored within the broader frame of child healthcare coverage and needs.

### Limitations

The quality of the primary data collection and reporting and the reliability of methods for prospectively estimating caseload or directly measuring caseload made it impossible to verify the true SAM population incidence. It was impossible to control for the possible effects of using different anthropometric indicators (WHZ or MUAC) that identified different SAM populations. Moreover, prevalence estimates covered different child populations by including or excluding infants 0–6 months of age. Prospective methods relied on a set of unrealistic assumptions (e.g. constant child populations, average duration of illness and prevalence adjusted for 50% coverage). The case study did not examine either the validity of the various methods and surveillance systems in accurately reflecting the magnitude or temporal and geographic variation of SAM, their cost-effectiveness or their sustainability. Instead, it was limited to applying figures and calculations used by health actors in Niger for planning, implementing and evaluating SAM interventions. The case study aimed to describe differences in results that were available and used in the country and their effect on planning. Therefore, comparing different SAM populations with gaps in some of the data weakened but did not change either the findings or the discussion.

## Conclusions

Reliable and accurate SAM caseload figures will continue to be in demand in Niger and elsewhere in the world. Indirect methods of estimating SAM caseload are inaccurate and rely on many unproven assumptions, yet continue to be used in the absence of direct incidence information. Prospective SAM caseload estimates derived from indirect methods should be used with great caution, as they considerably underestimate the true burden. The three surveillance systems in place in Niger either underestimated or overestimated true values of incidence, and causalities were intrinsically linked in complex ways. Differences in incidence numbers yielded by the systems caused confusion. Nevertheless, prospective caseload estimates and incidence numbers were used interchangeably for planning, implementation and evaluation of SAM services. Policy and service decisions that depend on these numbers may therefore be inappropriate and further weaken performance of service delivery [38].

Niger made major efforts to establish SAM surveillance systems for timely access to incidence information so that it could adapt resources to actual needs and maintain quality of services. Because of inadequate primary data measurement, reporting and recording methods or processes, it did not invest enough in ensuring the reliability of the incidence information. The country should consider improving SAM surveillance by improving primary data collection methods for easy and single entry of reliable data at the first contact with the health system. For example, anthropometry may be improved by using wide MUAC straps, length measures with infrared sensors and electronic tarred scales; medical recording may be improved by using mobile or smartphone or tablet applications linked with electronic health information systems. To be cost-effective, owned and sustainable, SAM surveillance should be aligned with the national health information system and be evaluated by systems methods that uncover the dynamics of the complex information system and the role of its actors [39]. Moreover, health actors in Niger may benefit from a health information exchange platform with open access that could disseminate the wealth of in-country learning and stimulate continuous learning in a fast changing context. Lessons from this case study may be relevant for countries with a high burden of acute malnutrition, including for targeted emergency responses.
